# Unregulated serving sizes on the Canadian nutrition facts table – an invitation for manufacturer manipulations

**DOI:** 10.1186/s12889-017-4362-0

**Published:** 2017-05-08

**Authors:** Jessica Yin Man Chan, Mary J. Scourboutakos, Mary R. L’Abbé

**Affiliations:** 1 Faculty of Medicine, Department of Nutritional Sciences, University of Toronto, Fitzgerald Building, 150 College St., Rm 315, Toronto, ON M1S 3E2 Canada; 2 Prenetics Ltd., Prenetics Ltd. 7/F, Prosperity Millennia Plaza, 663 King’s Rd, Quarry Bay, Hong Kong, China

**Keywords:** Nutrition facts table, Serving size, Standardized serving size, Reference amount, Nutrition labels, Nutrition labelling, Calorie density, Public health, Schedule M, Health Canada

## Abstract

**Background:**

Serving sizes on the Nutrition Facts table (NFt) on Canadian packaged foods have traditionally been unregulated and non-standardized. The federal government recently passed legislation to regulate the serving sizes listed on the NFt. The objective of this study was to compare the serving sizes on food product NFts to the recommendations in the 2003 Nutrition Labelling regulation (Schedule M) reference amounts, the Canadian Food Inspection Agency (CFIA) ranges, and Canada’s Food Guide recommendations. An additional objective was to determine if food and beverage products that report smaller serving sizes have a higher calorie density, compared to similar products with a larger serving size.

**Methods:**

Data for 10,487 products were retrieved from the 2010 Food Label Information Program (FLIP) database and categorized according to Schedule M categories. Correlations between calorie density and manufacturer stated serving size were tested and the proportion of products meeting recommendations were tabulated.

**Results:**

35% of products had serving sizes on the NFt that were smaller than the Schedule M reference amount and 23% exceeded the reference amount. 86% of products fell within the CFIA’s recommended serving size ranges; however, 70% were within the lower-half of the range. Several bread and juice categories exceeded CFG’s recommendations, while several dairy product categories were smaller than the recommendations. Of the 50 Schedule M sub-categories analyzed, 31 (62%) exhibited a negative correlation between serving size and calorie density.

**Conclusion:**

While most products fell within the CFIA’s recommended serving size ranges, there was a tendency for products with a higher calorie density to list smaller serving sizes.

**Electronic supplementary material:**

The online version of this article (doi:10.1186/s12889-017-4362-0) contains supplementary material, which is available to authorized users.

## Background

In recent years there has been a substantial increase in the prevalence of obesity in Canada. Presently 62.1% of Canadian adults are overweight, and 25% are obese [1]. The rise in obesity has been paralleled by the consumption of excess calories, partially due to increased portion sizes [2].

The Nutrition Facts table (NFt) is mandated to appear on nearly all packaged foods sold in Canada [3]. The serving size stated on the Nutrition Facts table determine the nutrient levels that will be reported on that label (for example, a smaller serving size reports fewer calories, while a larger serving size reports more calories). Traditionally, the serving sizes stated on the NFt on packaged foods sold in Canada were not standardized and could be determined by manufacturers, unlike in the United States, where the FDA regulates serving sizes [4]. Therefore, food companies could decide the serving size, and thus the number of calories a consumers sees when looking at a Nutrition Facts table. In other countries and jurisdictions, such as the EU, UK, and Australia, nutrition information is listed per 100 g to enable comparisons among similar products [5, 6]. The Canadian NFt does not feature nutrition information per 100 g.

Research has demonstrated that the reported serving sizes on NFts are often smaller than the portions typically consumed [7]. This suggests that food companies may be intentionally trying to reduce the reported calories on the nutrition label by using smaller serving sizes [8]. Additionally, research has demonstrated that using different serving sizes on the NFts of similar products, confuses consumers and makes comparisons among similar foods difficult. As a result, consumers have difficulty determining the energy content per serving and per package, and cannot accurately calculate calorie content when there is more than one serving per container [9]. Furthermore, anticipated guilt from consumption, purchase intentions, and choice behaviour, can be influenced by serving size manipulations, and may disproportionately influence weight-conscious consumers who are concerned about calories, but not serving size [8].

In Canada, two important government bodies i) Health Canada and ii) the Canadian Food Inspection Agency (CFIA) are responsible for Canadian food labelling regulations and public governance. Health Canada is responsible for administering the provisions of the Food and Drugs Act (FDR) that relate to public health, safety and nutrition [10]. Whereas the CFIA provides all federal inspection services related to food and enforces the food safety and nutritional quality standards established by Health Canada, i.e. responsible for the administration and enforcement of the Consumer Packaging and Labelling Act related to food [11]. The CFIA regulates the consistency, completeness and accuracy of the labelling and packaging of consumer goods. These regulations are intended to provide a fair and competitive marketplace by prohibiting deceptive labelling or advertising practices.

Reference amounts for the serving size on Nutrition Facts tables have been established by Health Canada and are set out in Schedule M of the Food and Drug Regulations (FDR) (B.01.001) [12]. Traditionally, these reference amounts were mandatory only as the basis for calculating the compositional criteria that manufacturers must meet for nutrient content claims and health claims [3]. For products without any nutrient content claims and health claims, the CFIA recommends manufacturers follow the range of serving sizes set in the CFIA Guide to Food Labelling and Advertising (CFIA guide), however, these ranges are not mandatory and only serves as a reference for manufacturers to stay within the recommended ranges [13]. In comparison, in the United States, standardized serving sizes used on the Nutrition Facts table have been regulated by the Food and Drug Administration (FDA) for more than 20 years and are required to conform to the Reference Amount Customarily Consumed (RACC) defined in section 101.12(b) of the food labelling regulations [14].

It was suggested that standardizing the serving sizes reported on the Canadian NFt could be an important policy intervention to help consumers make informed healthy food choices [3]. In December 2016, changes were made to the Food and Drug regulations in Canada that now require food manufacturers to use similar serving sizes for similar products [10, 15]. However, this new regulations will not be fully implemented until 2021. By modifying serving sizes to be more consistent and listing realistic measures, it is expected that Canadians will be more easily able to compare similar foods and make it easier to understand how many calories and nutrients they are consuming. This study was initiated before the new legislation was announced. Therefore, the objective of this study was to compare the serving sizes on food product NFTs to the recommendations in the 2003 Nutrition Labelling regulation (Schedule M) reference amounts, the CFIA recommended ranges, and Canada’s Food Guide (CFG) recommendations. The goal was to determine the number of foods that currently adhere to the voluntary Canadian FDR Schedule M serving size recommendations (reference amount) as well as the CFIA recommended serving ranges. Comparing serving sizes on food product NFTs to the CFG recommendations is needed to investigate the consistency between the serving sizes recommended in regulatory documents versus consumer education tools for healthy eating. Our second aim was to determine if food and beverage products that have a higher calorie density report a smaller serving size on the NFt, when compared to similar products with a larger serving size. Overall, the aim is for these results to shed light on the potential benefits of the new nutrition labelling changes, to be implemented on Canada, over the next five years.

## Methods

### Data collection

This was a cross-sectional analyses of the serving size and calories listed on the NFt on 10,487 packaged foods from Canadian grocery stores. Canadian food package label information, as reported on the NFt, was retrieved from the 2010 Food Label Information Program (FLIP) database at the University of Toronto [16]. All data were collected between March 2010 and April 2011 from outlets of the four largest grocery chains in Canada (Loblaws, Metro and Sobeys in Ontario) and one major western Canadian grocery retailer (Safeway, in Alberta). These chains represented approximately 75% of the market share of grocery food products sold in Canada; therefore, most national and private label branded food products were collected. A total of 10,487 unique food products were in the FLIP database. Additional details concerning the construction of the FLIP 2010 database can be found elsewhere [16].

### Food classifications and reference serving sizes

All food items were categorized according to the Schedule M categories and sub-categories, as described in the Canadian Food Inspection Agency (CFIA) guide. Schedule M is a component of the Canadian Food and Drug Nutrition Labelling Regulations [B.01.001] and lists serving size reference amounts and recommended serving size ranges for 22 categories and 153 subcategories [3]. The reference amount is a specific regulated quantity of food (measured in grams) and it is meant to represent the portion that would typically be eaten by an individual at one sitting, but is not required to be used by manufacturers on the NFt (Additional file 1).

The CFIA guide provides a range of suggested serving sizes within each of the Schedule M subcategories to guide manufacturer determined serving sizes [3]. The ranges are meant to give manufacturers flexibility when determining the appropriate serving size to disclose on a product’s NFt, however, manufacturers are not required to follow these serving size reference amounts. Use of reference amounts are only mandatory as the basis for determining eligibility of a food to carry nutrient content claims and health claims.

In order to compare manufacturer stated serving sizes to a standardized serving size, schedule M reference amounts were assigned to each food product based on the sub-category that best matched the product’s description. To ensure that food items were categorized consistently, data were checked by a second independent reviewer. In any case of discrepancies, the CFIA was contacted to verify categorizations. A description of the Schedule M subcategories and the food products within each subcategory can be found in Additional file 1.

### Inclusion and exclusion criteria

Of the 153 schedule M sub-categories, all sub-categories with at least 50 unique food items were included in this analysis leaving a total of 7494 foods for analysis. For categories with less than 50 food items, sample size might be too small to reflect all existing products across the country. Thus, excluding those categories might help reduce selection bias.

#### Data analysis

Descriptive statistics were calculated for the serving size and calorie content listed on the NFt (according to the manufacturer stated serving size). The proportion of products with serving sizes that were less than, equal to, or greater than the reference amount listed in Schedule M were tabulated. The proportions of products with serving sizes below, within, and above the range of recommended serving sizes set out in the CFIA Guide were also tabulated. Additionally, when a product’s serving size was within the CFIA range of recommended serving sizes, the proportion of products in the lower half and upper half of the range was calculated.

Each product’s calorie density (calculated as calories per 100 g and calories per reference amount) were calculated. For each product, scatter plots for the calories per reference amount in comparison to the food product’s stated serving size were created to study the association between calorie density and serving size. Correlations between calorie density and serving size were tested using Pearson correlation.

The sign test was used to detect differences between the calories per serving and calories per reference amount, within each food category. All statistical analyses were performed using Statistica, version 10 (Tulsa, OK). A *p*-value <0.05 was considered significant.

## Results

Fifty schedule M sub-categories containing 7494 food products were analysed. The 50 sub-categories included in this study are listed in Table 1.

**Table 1:** The proportion of products with manufacturer stated serving sizes that are equal to, less than, or greater than the Schedule M reference amounts and recommended ranges

Food group	Number	Schedule M Reference Amounts^a^			CFIA Recommended Serving Size Ranges^b^				
		< reference amount	═ reference amount	> reference amount	< recommended serving size	within recommended serving size	> recommended serving size	Among products within the recommended range	
								Lower-half	Upper-half
Bakery Products									
1. Bread	183	67 (37%)	15 (8%)	101 (55%)	2 (1%)	135 (74%)	46 (25%)	65 (48%)	70 (52%)
2. Bagels, tea biscuits, scones etc.	227	84 (37%)	6 (3%)	137 (60%)	3 (1%)	221 (98%)	3 (1%)	123 (56%)	98 (44%)
7. Coffee cakes, doughnuts, danishes etc.	89	42 (47%)	6 (7%)	41 (46%)	27 (30%)	57 (64%)	5 (6%)	55 (96%)	2 (4%)
8. Cookies, graham wafers	294	116 (39%)	63 (22%)	115 (39%)	116 (40%)	166 (56%)	12 (4%)	139 (84%)	27 (16%)
9. Crackers, hard bread sticks etc.	238	54 (23%)	83 (35%)	101 (42%)	7 (3%)	220 (92%)	11 (5%)	184 (84%)	36 (16%)
14. Croutons	53	0 (0%)	41 (77%)	12 (23%)	0 (0%)	51 (96%)	2 (4%)	46 (90%)	5 (10%)
15. French toast, pancakes, and waffles	93	87 (94%)	5 (5%)	1 (1%)	0 (0%)	92 (99%)	1 (1%)	67 (73%)	25 (27%)
17. Grain-based bars with filling and coating	85	38 (45%)	20 (23%)	27 (32%)	0 (0%)	85 (100%)	0 (0%)	79 (93%)	6 (7%)
18. Rice cakes and corn cakes	62	39 (63%)	2 (3%)	21 (34%)	2 (3%)	54 (87%)	6 (10%)	39 (73%)	15 (27%)
19. Pies, tarts, cobblers, turnovers	94	66 (70%)	6 (6%)	22 (24%)	16 (17%)	77 (82%)	1 (1%)	35 (45%)	42 (55%)
Cereals and Other Grain Products									
28. Hot breakfast cereals	57	32 (56%)	5 (9%)	20 (35%)	4 (7%)	33 (58%)	20 (35%)	12 (36%)	21 (64%)
30. Breakfast cereals without fruit or nuts	85	19 (22%)	51 (60%)	15 (18%)	0 (0%)	85 (100%)	0 (0%)	78 (93%)	7 (7%)
31. Breakfast cereals with fruit and nuts	145	52 (36%)	66 (46%)	27 (18%)	26 (18%)	119 (82%)	0 (0%)	119 (100%)	0 (0%)
34. Grains, such as rice or barley	85	10 (12%)	44 (52%)	31 (36%)	0 (0%)	54 (64%)	31 (36%)	7 (13%)	47 (87%)
35. Pastas without sauce	383	58 (15%)	290 (76%)	35 (9%)	7 (2%)	376 (98%)	0 (0%)	37 (10%)	339 (90%)
Dairy Products and Substitutes									
39. Cheese	380	106 (28%)	246 (65%)	28 (7%)	23 (6%)	357 (94%)	0 (0%)	352 (99%)	5 (1%)
43. Quark, fresh cheese and fresh dairy desserts	63	62 (98%)	1 (2%)	0 (0%)	57 (90%)	6 (10%)	0 (0%)	6 (100%)	0 (0%)
49. Plant-based beverages	138	33 (24%)	102 (74%)	3 (2%)	5 (4%)	130 (94%)	3 (2%)	3 (2%)	127 98%)
52. Yogurt	95	68 (72%)	27 (38%)	0 (0%)	30 (32%)	65 (68%)	0 (0%)	65 (100%)	0 (0%)
Desserts									
53. Ice cream, ice milk, frozen yogurt, sherbet	282	11 (4%)	269 (95%)	2 (1%)	0 (0%)	282 (100%)	0 (0%)	282 (100%)	0 (0%)
54. Dairy desserts, frozen	97	78 (80%)	6 (6%)	13 (14%)	15 (15%)	82 (85%)	0 (0%)	55 (67%)	27 (33%)
Fats and Oils									
64. Butter, margarine, shortening, lard	91	2 (2%)	88 (97%)	1 (1%)	0 (0%)	91 (100%)	0 (0%)	91 (100%)	0 (0%)
65. Vegetable oil	105	0 (0%)	82 (78%)	23 (22%)	0 (0%)	105 (100%)	0 (0%)	82 (78%)	23 (22%)
67. Dressings for salad	227	209 (92%)	18 (8%)	0 (0%)	0 (0%)	227 (100%)	0 (0%)	209 (92%)	18 (8%)
Marine and Fresh Water Animals									
72. Marine and fresh water animals	132	94 (71%)	13 (10%)	25 (19%)	15 (12%)	94 (71%)	23 (17%)	94 (100%)	0 (0%)
73. Marine and fresh water animals, canned	116	11 (9%)	16 (14%)	89 (77%)	1 (1%)	106 (91%)	9 (8%)	65 (61%)	41 (39%)
Fruits and Fruit Juices									
75. Fruit, fresh, canned or frozen	168	129 (77%)	9 (5%)	30 (18%)	7 (4%)	161 (96%)	0 (0%)	122 (76%)	39 (24%)
77. Dried fruit	69	10 (14%)	47 (68%)	12 (18%)	4 (6%)	53 (77%)	12 (17%)	4 (8%)	49 (92%)
83. Juices, nectars and fruit drinks	554	155 (28%)	399 (72%)	0 (0%)	82 (15%)	472 (85%)	0 (0%)	63 (13%)	409 (87%)
Legumes									
86. Beans, peas and lentils	78	27 (35%)	50 (64%)	1 (1%)	0 (0%)	77 (99%)	1 (1%)	21 (27%)	56 (73%)
Meat, Poultry, Their Products and Substitutes									
90. Luncheon meats	107	26 (24%)	24 (23%)	57 (53%)	0 (0%)	105 (98%)	2 (2%)	24 (23%)	81 (77%)
91. Sausages	102	36 (35%)	9 (9%)	57 (56%)	0 (0%)	98 (96%)	4 (4%)	59 (60%)	39 (40%)
93. Patties, cutlettes, chopettes etc.	103	14 (14%)	27 (26%)	62 (60%)	5 (5%)	65 (63%)	33 (32%)	39 (60%)	26 (40%)
96. Meat and poultry with sauce	106	80 (75%)	12 (12%)	14 (13%)	6 (6%)	92 (87%)	8 (7%)	54 (59%)	38 (41%)
Miscellaneous category									
99. Bread crumbs and batter mixes	151	22 (15%)	12 (8%)	117 (77%)	3 (2%)	145 (96%)	3 (2%)	56 (39%)	89 (61%)
Combination Dishes									
107. Measurable	366	175 (48%)	27 (7%)	164 (45%)	76 (21%)	282 (77%)	8 (2%)	218 (77%)	64 (23%)
108. Not measurable	304	144 (48%)	13 (4%)	147 (48%)	25 (8%)	266 (88%)	13 (4%)	223 (84%)	43 (16%)
109. Hor d’oeuvres	104	39 (38%)	6 (6%)	59 (56%)	2 (2%)	74 (71%)	28 (27%)	57 (77%)	17 (23%)
Nuts and Seeds									
110. Nuts and seeds	67	3 (4%)	24 (36%)	40 (60%)	3 (4%)	64 (96%)	0 (0%)	64 (100%)	0 (0%)
Sauces, Dips, Gravies and Condiments									
120. Sauces for dipping	117	2 (2%)	112 (96%)	3 (2%)	0 (0%)	114 (97%)	3 (3%)	114 (100%)	0 (0%)
121. Dips	92	36 (39%)	18 (20%)	38 (41%)	1 (1%)	91 (99%)	0 (0%)	82 (90%)	9 (10%)
122. Major main entree sauce	145	20 (14%)	125 (86%)	0 (0%)	11 (8%)	134 (92%)	0 (0%)	134 (100%)	0 (0%)
123. Minor main entree sauce	102	39 (38%)	55 (54%)	8 (8%)	36 (35%)	63 (62%)	3 (3%)	61 (97%)	2 (3%)
124. Major condiments	100	1 (1%)	97 (97%)	2 (2%)	0 (0%)	100 (100%)	0 (0%)	98 (98%)	2 (2%)
125. Minor condiments	48	0 (0%)	45 (94%)	3 (6%)	0 (0%)	45 (94%)	3 (6%)	45 (100%)	0 (0%)
Snacks									
126. Chips, pretzels, popcorn, extruded snacks	375	147 (39%)	228 (61%)	0 (0%)	82 (22%)	293 (78%)	0 (0%)	293 (100%)	0 (0%)
127. Nuts or seeds for use as snacks	88	19 (22%)	66 (75%)	3 (3%)	3 (3%)	82 (94%)	3 (3%)	82 (100%)	0 (0%)
Sugars and Sweets									
137. Jams, jellies, marmalades etc.	145	0 (0%)	143 (99%)	2 (1%)	0 (0%)	143 (99%)	2 (1%)	143 (100%)	0 (0%)
141. syrups	50	34 (68%)	15 (30%)	1 (2%)	9 (18%)	40 (80%)	1 (2%)	25 (63%)	15 (37%)
Vegetables									
150. Pickles	54	20 (37%)	28 (52%)	6 (11%)	N/A	N/A	N/A	N/A	N/A
All Food Groups	7494	2616 (35%)	3162 (42%)	1716 (23%)	756 (10%)	6429 (86%)	300 (4%)	4470 (70%)	1959 (30%)

^a^Schedule M of the Nutrition Labelling regulations^3^ lists reference amounts that are used as the basis for determining qualifying/disqualifying criteria for nutrient content claims and health claims. Their use on the Nutrition Facts table is not mandatory and manufacturers can determine the serving size used on the Nutrition Facts table

^b^The CFIA guide suggests recommended serving size ranges for each Schedule M food category, but manufacturer’s adherence to these ranges is not mandatory [13]

### Comparison of serving sizes in relation to schedule M reference amounts

Table 1 compares the manufacturer stated serving sizes reported on the NFt with the Schedule M reference amount and CFIA recommended serving size ranges. 35% of products had serving sizes that were lower than the reference amount in schedule M, 42% of products had serving sizes that were consistent with the reference amount, and 23% exceeded the reference amount. In nine categories, (representing 18% of all categories) more than 70% of products had serving sizes that were smaller than the reference amount. The nine categories were ‘French toast, pancakes, and waffles’; ‘Pies, tarts, cobblers, turnovers’; ‘Quark, fresh cheese and fresh dairy desserts’; ‘Yogurt’; ‘Dairy desserts, frozen’; ‘Dressings for salad’; ‘Marine and fresh water animals’; ‘Fruit, fresh, canned or frozen’ and ‘Meat and poultry with sauce’. Furthermore, in an additional twelve categories, (representing 24% of all categories) more than half of the products had serving sizes that were smaller than the reference amount.

### Comparison of serving sizes in relation to CFIA recommended serving size ranges

When compared to the CFIA recommended serving size ranges, 10% of products had manufacturer stated serving sizes that were smaller than the recommended range, 86% were within the recommended range, and 4% were larger than the recommended range. However, among products whose serving size fell within the recommended range, 70% fell within the lower-half of the recommended range, while 30% fell within the higher half of the recommended range.

### Comparison of serving sizes in relation to Canada’s food guide

Only a limited number of categories could be compared to the Canada Food Guide recommended serving sizes due to different food categorization systems. In addition, the recommended serving sizes in CFG were primarily based on cooked food portions, whereas the serving size on food product NFts were based on raw food portions, this further limited the number of categories that could be compared. In ‘bread’; ‘bagels, tea biscuits, scones’; and ‘juices, nectars and fruit drinks’, 80–90% of products had manufacturer stated serving sizes that were higher than those recommended by Canada’s food guide. Meanwhile in ‘cheese’ and ‘quark, fresh cheese and fresh dairy desserts’, 90–99% of products had manufacturer stated serving sizes that were smaller than the Canada Food Guide Recommended Serving Sizes.

### Comparison of calorie content currently listed on the NFt versus the reference amount

Table 2 shows the median calories in each category as currently listed on the NFt in comparison to the amount that would be listed if manufacturers were required to use the reference amounts for standardized serving sizes. In 21 of the 50 categories analysed (42% of categories), the median amount of calories reported on the NFt (for that category) was significantly lower than the amount that would be stated if manufacturers were required to adhere to the reference amount serving sizes. In contrast, there were only seven categories (14% of categories) where the median calories based on the manufacturer stated serving size was significantly higher than the amount of calories that would be stated if reference amounts were required. Notably ‘Quark, fresh cheese and fresh dairy desserts’; ‘Dressings for salad’ and ‘Syrups’ had the highest differences in calorie levels which would be stated 317%, 200% and 183% larger, respectively, if the standardized reference amounts were used on the NFt. Categories showing a moderately low manufacturer stated serving size (20% to 30%, when compared to schedule M reference amounts) included: ‘Yogurt’; ‘Marine and fresh water animals’; ‘Meat and poultry with sauce’; and ‘Minor main entree with sauce’.

**Table 2:** Comparison between the median calories currently listed on the Nutrition Facts Table (NFT) and the calories per reference amount

Food group	Number	Median Calories (kcal)/manufacturer stated serving size on NFT (g)	Median Calories (kcal)/reference amount (g)	p*	Minimum calories (kcal)/serving size on NFT† (g)	Maximum calories (kcal)/serving size on NFT^a^ (g)
Bakery Products						
1. Bread	183	130	127	0.011	60	230
2. Bagels, tea biscuits, scones etc.	227	150	147	0.001	40	350
7. Coffee cakes, doughnuts, danishes etc.	89	200	215	0.913	60	520
8. Cookies, graham wafers	294	140	141	0.999	30	250
9. Crackers, hard bread sticks etc.	238	90	90	na	60	247
14. Croutons	53	35	30	0.001	25	110
15. French toast, pancakes, and waffles	93	140	167	0.001^‡^	90	230
17. Grain-based bars with filling and coating	85	110	123	0.215	90	230
18. Rice cakes and corn cakes	62	60	64	0.028^‡^	30	230
19. Pies, tarts, cobblers, turnovers	94	305	321	0.001^‡^	80	430
Cereals and Other Grain Products						
28. Hot breakfast cereals	57	150	155	0.127	90	210
30. Breakfast cereals without fruit or nuts	85	120	116	0.607	80	130
31. Breakfast cereals with fruit and nuts	145	210	214	0.022^‡^	90	270
34. Grains, such as rice or barley	85	160	160	0.349	110	360
35. Pastas without sauce	383	300	302	0.007^‡^	110	342
Dairy Products and Substitutes						
39. Cheese	380	90	100	0.001^‡^	20	190
43. Quark, fresh cheese and fresh dairy desserts	63	90	286	0.001^‡^	60	190
49. Plant-based beverages	138	120	130	0.001^‡^	30	230
52. Yogurt	95	100	140	0.001^‡^	35	260
Desserts						
53. Ice cream, ice milk, frozen yogurt, sherbet	282	140	140	na	60	340
54. Dairy desserts, frozen	97	180	250	0.001	40	360
Fats and Oils						
64. Butter, margarine, shortening, lard	91	70	70	na	25	90
65. Vegetable oil	105	80	80	0.001^§^	80	130
67. Dressings for salad	227	45	90	0.001^‡^	10	160
Marine and Fresh Water Animals						
72. Marine and fresh water animals	132	170	223	0.001^‡^	65	540
73. Marine and fresh water animals, canned	116	90	71	0.001	25	240
Fruits and Fruit Juices						
75. Fruit, fresh, canned or frozen	167	80	91	0.001^‡^	30	220
77. Dried fruit	69	120	122	0.831	40	270
83. Juices, nectars and fruit drinks	553	120	125	0.001^‡^	10	200
Legumes						
86. Beans, peas and lentils	78	340	350	0.001^‡^	35	420
Meat, Poultry, Their Products and Substitutes						
90. Luncheon meats	107	60	61	0.001^‡^	30	170
91. Sausages	102	150	142	0.03	40	370
93. Patties, cutlettes, chopettes etc.	103	230	211	0.001	70	550
96. Meat and poultry with sauce	106	190	252	0.001^‡^	90	410
Miscellaneous category						
99. Bread crumbs and batter mixes	151	150	118	0.001	30	350
Combination Dishes						
107. Measurable	366	275	284	0.957	110	700
108. Not measurable	304	290	309	0.907	80	660
109. Hor d’oeuvres	104	155	124	0.055	70	452
Nuts and Seeds						
110. Nuts and seeds	67	260	190	0.001	80	380
Sauces, Dips, Gravies and Condiments						
120. Sauces for dipping	117	60	60	na	10	170
121. Dips	92	60	63	0.907	15	170
122. Major main entree sauce	145	70	70	0.001^§^	20	270
123. Minor main entree sauce	102	20	25	0.001^‡^	10	310
124. Major condiments	100	20	20	na	5	70
125. Minor condiments	48	5	5	0.248	0	80
Snacks						
126. Chips, pretzels, popcorn, extruded snacks	375	240	250	0.001^‡^	40	330
127. Nuts or seeds for use as snacks	88	280	290	0.021^‡^	160	440
Sugars and Sweets						
137. Jams, jellies, marmalades etc.	145	50	50	0.479	5	80
141. Syrups	50	120	220	0.001^‡^	30	468
Vegetables						
150. Pickles	54	10	9	0.025	3	70

^a^ *NFT* = Nutrition Facts Table, the mandatory nutrition labelling required on all packaged food products

*because data was non-normal, *p*-values reflect significance according to the Sign Test

^‡^indicates categories where the median calories per reference amount is significantly greater than the median calories per stated serving

^§^In two instances the *p*-value is significant but there is no difference in the median, this is due to the fact that signficance was determined according to the sign-test

### Correlation between calorie density and serving size

There was a significant negative correlation between serving size and calorie density in 31 categories (62% of categories) (Table 3). In 22 of these categories (44%), the negative correlation was significant (*p* < 0.05). ‘Juices, nectars and fruit drinks’ showed the strongest negative correlation (−0.9, *p* < 0.0001) while ‘Croutons’, ‘Quark, fresh cheese and fresh dairy desserts’, ‘Plant-based beverages’, ‘Measurable combination dishes’, ‘Not measurable combination dishes’ and ‘Major main entree with sauce’ also showed a significant negative correlations ranging from −0.4 to −0.6 (*p* < 0.001).

**Table 3:** Correlation between serving size and calorie density in each Schedule M^a^ food category

Food group	Number	Pearson Correlation - r	*p*
Bakery Products			
1. Bread	183	−0.3493	0
2. Bagels, tea biscuits, scones etc.	227	0.006	0.0001
7. Coffee cakes, doughnuts, danishes etc.	89	−0.3107	0.003
8. Cookies, graham wafers	294	−0.0972	0.0961
9. Crackers, hard bread sticks etc.	238	−0.2227	0.0005
14. Croutons	53	−0.6452	0
15. French toast, pancakes, and waffles	93	−0.2151	0.0384
17. Grain-based bars with filling and coating	85	0.1998	0.0667
18. Rice cakes and corn cakes	62	0.5732	0
19. Pies, tarts, cobblers, turnovers	94	−0.3318	0.0011
Cereals and Other Grain Products			
28. Hot breakfast cereals	57	0.2337	0.0802
30. Breakfast cereals without fruit or nuts	85	−0.3751	0.0004
31. Breakfast cereals with fruit and nuts	145	0.0097	0.9079
34. Grains, such as rice or barley	85	0.2446	0.0241
35. Pastas without sauce	383	−0.008	0.8757
Dairy Products and Substitutes			
39. Cheese	380	0.2066	0.00005
43. Quark, fresh cheese and fresh dairy desserts	63	−0.4323	0.0004
49. Plant-based beverages	138	−0.4618	0
52. Yogurt	95	−0.0726	0.4845
Desserts			
53. Ice cream, ice milk, frozen yogurt, sherbet	282	0.2197	0.0002
54. Dairy desserts, frozen	97	0.5856	0
Fats and Oils			
64. Butter, margarine, shortening, lard	91	−0.202	0.0548
65. Vegetable oil	105	0.2112	0.0306
67. Dressings for salad	227	0.0552	0.408
Marine and Fresh Water Animals			
72. Marine and fresh water animals	132	−0.353	0.00003
73. Marine and fresh water animals, canned	116	0.2288	0.0135
Fruits and Fruit Juices			
75. Fruit, fresh, canned or frozen	167	−0.2265	0.0032
77. Dried fruit	69	−0.2073	0.0874
83. Juices, nectars and fruit drinks	553	−0.9073	0
Legumes			
86. Beans, peas and lentils	78	−0.227	0.0457
Meat, Poultry, Their Products and Substitutes			
90. Luncheon meats	107	0.1014	0.2989
91. Sausages	102	−0.0376	0.0014
93. Patties, cutlettes, chopettes etc.	103	0.1431	0.1493
96. Meat and poultry with sauce	106	−0.3741	0.00008
Miscellaneous category			
99. Bread crumbs and batter mixes	151	−0.2137	0.0084
Combination Dishes			
107. Measurable	366	−0.5304	0
108. Not measurable	304	−0.6052	0
109. Hor d’oeuvres	104	−0.2401	0.0141
Nuts and Seeds			
110. Nuts and seeds	67	−0.2491	0.0421
Sauces, Dips, Gravies and Condiments			
120. Sauces for dipping	117	−0.2977	0.0011
121. Dips	92	−0.0984	0.3506
122. Major main entree sauce	145	−0.4883	0
123. Minor main entree sauce	102	0.2153	0.0298
124. Major condiments	100	0.2593	0.0092
125. Minor condiments	48	0.1166	0.43
Snacks			
126. Chips, pretzels, popcorn, extruded snacks	375	0.5575	0
127. Nuts or seeds for use as snacks	88	−0.1716	0.1099
Sugars and Sweets			
137. Jams, jellies, marmalades etc.	145	0.0318	0.7042
141. syrups	50	−0.0321	0.8323
Vegetables			
150. Pickles	54	−0.1669	0.2278

50 of the 153 categories in schedule M had greater than 50 foods and thus were included in the analysis

^a^Schedule M is a component of the Food and Drug Regulations (B.01.001) which includes reference amounts and recommended serving sizes for the Nutrition Facts table on packaged food products. These references are voluntary and are only mandatory when manufacturers are aiming to meet the compositional criteria for nutrient content claims and health claims

## Discussion

This study demonstrates that 35% of Canadian food products had manufacturer stated serving sizes that were lower than the Schedule M reference amount. While many products fell within the CFIA recommended serving size ranges (which are quite large), the majority (70%) were within the lower half of the range. Furthermore, in the majority of food categories, products with a smaller manufacturer stated serving size tended to have a higher calorie density. Therefore, the lack of regulated serving sizes on the NFt on packaged foods in Canada has led to a tendency for food manufacturers to state smaller serving sizes and consequently display lower calorie levels, particularly in high calorie density foods. Collectively, these results suggest that there is an urgent need to regulate and standardize the NFt serving sizes, as the current unregulated system has led to a large proportion of food products with a higher calorie density to report smaller serving sizes, which can be misleading to consumers.

These findings are concerning because it has been shown that only knowledgeable consumers will be motivated to spend time analyzing nutrition information accurately and few are able to do the calculations necessary to compare products with different serving sizes [13]. These results also illustrated the very wide range of serving sizes (some as high as ten-fold) within categories, used by manufacturers in Canada. Health Canada consumer research has shown that consumers find it difficult to compare products, particularly when different serving sizes are used on the Nft [13]; thus consumers may be falsely led to believe that they are consuming fewer calories, when in fact, they are simply eating less food. Data illustrate that the current non-standardized serving size system in Canada is confusing and can lead to dramatic underestimation of calorie intakes [7, 17]. Additionally, this is worrisome, because research has highlighted that certain consumers, such as those who are sensitive to potentially negative nutrients (such as calories), as well as those with less knowledge of nutrition, are likely to be most susceptible to serving size manipulations [18].

This study also illustrates the need to update the serving size recommendations and ranges outlined in Schedule M, to be more in line with the serving size recommendations in Canada’s Food Guide. For example, in the ‘juices, nectars and fruit drinks’ category, most of the product’s serving sizes were in agreement with the reference amount, yet greater than 84% of products exceeded the recommended serving size in CFG. This finding illustrates the disparities between the serving sizes recommended in regulatory documents versus consumer education tools for healthy eating. Therefore, while schedule M and the CFIA make their recommendations based on what is typically consumed, this may not reflect what is recommended in Canada’s Food Guide. Using the amount typically consumed, rather than the recommended serving sizes, as the criteria for labelling, may in fact, promote increased serving sizes and food intakes and contribute to the increasing rate of obesity in Canada.

Standardizing serving sizes as well as aligning them with recommended servings in Canada’s Food Guide, is only one potential solution to this problem. For example, in the food regulations set out by the Food Standards Australia New Zealand, products are required to present nutrient levels both per serving size and per 100 g/mL using a dual-column system, thus enabling comparisons amongst products irrespective of their serving sizes [17]. The EU similarly avoids the need to regulate serving sizes by reporting nutrient levels per 100 g [19]. Interestingly, the “Labelling Logic Review” in Australia, recommended that serving sizes be removed from the Nutrition Information Panel (NIP), aiming to simplify requirements for the mandatory NIP and reduce the regulatory burden on industry [20]. However, no further work has been be undertaken on this recommendation due to the perceived lack of benefit [21]. The Public Health Association of Australia (PHAA) stated that removing the serving size column would not solve the problem of consumer confusion and recommended that the only approach to dealing with the inconsistency in serving sizes is to mandate serving sizes within food categories, as is being currently implemented in Canada [15].

Furthermore, the more fundamental question is, what types of nutrition label information actually assists consumers to make healthier food choices? For example, *Roberto and Khandpur* had suggested package design might also help educate consumers about appropriate serving sizes by having markers on the outside of food packaging that denote serving size amounts; or having clear indicators of pre-portioned servings in the package design [22]. Not to mention, effective consumer education is an essential co-requirement to enable consumers to understand the valuable information on the NFt.

This study evaluated a large number of foods from a wide variety of food categories. Limitations include the fact that Schedule M serving sizes were not available for a number of sub-categories. In addition, our study only investigated calories, and did not analyze other nutrient levels in relation to the manufacturer stated serving size. Hunter et al. noted that discrepancies in serving-size are often attributed to the use of food products for different purposes [23], thus a higher serving size could be advantageous if the manufacturer inflates the content of a healthy nutrient. Our study did not investigate other factors that could motivate serving size manipulations.

## Conclusion

These findings provide data to support the benefits of standardized serving sizes on Nutrition Facts tables. The study also reinforces findings from previous research studies which suggest that in jurisdictions where serving sizes are not standardized, manufacturers can alter consumer perceptions of the healthfulness of a product—particularly its calorie level—simply by decreasing the serving size, without changing the overall nutritional quality of the product, as illustrated by the negative correlation between serving size and calorie density. Therefore, in light of the obesity and diet-related chronic disease epidemic, further research is required to inform policies to help consumers make sense of the NFt amidst a confusing food environment. Nutrition labelling policies that assist consumers to make informed food selection choices are one step towards addressing this pressing public health issue.

## Additional file

Additional file 1: List of food categories with reference amount, serving size and FLIP data characteristics. This table outlines the food categories included in the paper along with the Schedule M reference amounts, CFIA suggested serving sizes and the number of products in the FLIP database that corresponded to each category. (DOCX 23 kb)

## Abbreviations

CFG: Canada’s Food Guide
CFIA: Canadian Food Inspection Agency
EU: European Union
FLIP: Food Label Information Program
FDA: Food and Drug Administration
FDR: Food and Drug Regulations
NIP: Nutrition Information Panel
NFt: Nutrition Facts table
PHAA: Public Health Association of Australia
RACC: Reference Amount Customarily Consumed
UK: United Kingdom
