# Supplementary material for: Unregulated serving sizes on the Canadian nutrition facts table – an invitation for manufacturer manipulations
Source: BMC Public Health. 2017 May 8;17:418. doi: 10.1186/s12889-017-4362-0 (PMC5423016; doi:10.1186/s12889-017-4362-0)
Supplement: Additional file 1: — List of food categories with reference amount, serving size and FLIP data characteristics. This table outlines the food categories included in the paper along with the Schedule M reference amounts, CFIA suggested serving sizes and the number of products in the FLIP database that corresponded to each category. (DOCX 23 kb) [file 12889_2017_4362_MOESM1_ESM.docx]

| **Additional File 1** List of food categories with reference amount, serving size and FLIP data characteristics |  |  |  |  |
| --- | --- | --- | --- | --- |
| **Food group** | **Remarks** | **Reference amount (g)** | **CFIA suggested serving size range (g)** | **No. of products in FLIP 2010** |
| **Bakery Products** |  |  |  |  |
| 1. Bread | Excluding sweet quick-type rolls | 50 | 25-70 | 183 |
| 2. Bagels, tea biscuits, scones etc. | + rolls, buns, croissants, tortillas, soft bread sticks, soft pretzels and corn bread | 55 | 25-100 | 227 |
| 7. Coffee cakes, doughnuts, danishes etc. | + sweet rolls, sweet quick-type breads and muffins | 55 | 50-100 | 89 |
| 8. Cookies, graham wafers | with or without coating or filling | 30 | 30-40 | 294 |
| 9. Crackers, hard bread sticks etc. | + melba toast | 20 | 15-30 | 238 |
| 14. Croutons |  | 7 | 7 – 20 | 53 |
| 15. French toast, pancakes, and waffles |  | 40 | 20-50 | 93 |
| 17. Grain-based bars with filling and coating |  | 30 | 20-50 | 85 |
| 18. Rice cakes and corn cakes |  | 15 | 10 – 25 | 62 |
| 19. Pies, tarts, cobblers, turnovers | + other pastries | 110 | 85-120 | 94 |
| **Cereals and Other Grain Products** |  |  |  |  |
| 28. Hot breakfast cereals | such as oatmeal, or cream of wheat | 40 | 30-40 | 57 |
| 30. Breakfast cereals without fruit or nuts | puffed and coated, flaked, extruded | 30 | 20-45 | 85 |
| 31. Breakfast cereals with fruit and nuts | + granola and biscuit type cereals | 55 | 45-80 | 145 |
| 34. Grains, such as rice or barley |  | 45 | 30-45 | 85 |
| 35. Pastas without sauce |  | 85 | 45-100 | 383 |
| **Dairy Products and Substitutes** |  |  |  |  |
| 39. Cheese | including cream cheese and cheese spread, except those listed as a separate item | 30 | 15-60 | 380 |
| 43. Quark, fresh cheese and fresh dairy desserts | | 100 | 50-200 | 63 |
| 49. Plant and milk-based beverages |  | 250 | 125-250 | 138 |
| 52. Yogurt |  | 175 | 125-225 | 95 |
| **Desserts** |  |  |  |  |
| 53. Ice cream, ice milk, forzen yogurt, sherbet | | 125 | 60-250 | 282 |
| 54. Dairy desserts, frozen | such as cakes, bars, sandwiches or cones | 125 | 60-175 | 97 |
| **Fats and Oils** |  |  |  |  |
| 64. Butter, margarine, shortening, lard |  | 10 | 5 – 20 | 91 |
| 65. Vegetable Oil |  | 10 | 5 – 20 | 105 |
| 67. Dressings for salad |  | 30 | 15-30 | 227 |
| **Marine and Fresh Water Animals** |  |  |  |  |
| 72. Marine and fresh water animals | such as plain or fried fish or shellfish, or fish or shellfish cakes | 125 | 85-130 | 132 |
| 73. Marine and fresh water animals, canned |  | 55 | 50-100 | 116 |
| **Fruits and Fruit Juices** |  |  |  |  |
| 75. Fruit, fresh, canned or frozen |  | 140 | 110-160 | 167 |
| 77. Dried fruit | such as raisins, dates or figs | 40 | 30-40 | 69 |
| 83. Juices, nectars and fruit drinks |  | 250 | 175-250 | 553 |
| **Legumes** |  |  |  |  |
| 86. Beans, peas and lentils | such as white beans, kidney beans, romano beans, soybeans or chick peas | 100 | 35-100 | 78 |
| **Meat, Poultry, Their Products and Substitutes** | |  |  |  |
| 90. Luncheon meats | such as bologna, blood pudding, minced luncheon roll, liver sausage, mortadella | 55 | 25-75 | 107 |
| 91. Sausages | such as linked sausage, Vienna sausage, wieners, breakfast sausage, franfurters | 55 | 25-115 | 102 |
| 93. Patties, cutlettes, chopettes etc. | + steakettes, meatballs, sausage meat and ground meat | 100 | 80-130 | 103 |
| 96. Meat and poultry with sauce | such as meat in barbecue sauce or turkey with gravy | 140 | 90-150 | 106 |
| **Miscellaneous category** |  |  |  |  |
| 99. Bread crumbs and batter mixes |  | 30 | 15-60 | 151 |
| **Combination Dishes** |  |  |  |  |
| 107. Measurable | such as casserole, hash, macroni and cheese with or without meat, pot pie | 250 | 200-375 | 366 |
| 108. Not measurable | such as burritos, egg rolls, enchiladas, pizza, pizza rolls, sausage rolls, pastry rolls | 140 | 90-300 | 304 |
| 109. Hor d'oeuvres |  | 50 | 25-100 | 104 |
| **Nuts and Seeds** |  |  |  |  |
| 110. Nuts and seeds | not fur use as snacks: whole, chopped, sliced, silvered or ground | 30 | 30-75 | 67 |
| **Sauces, Dips, Gravies and Condiments** |  |  |  |  |
| 120. Sauces for dipping | such as barbecue, hollandaise, tartar, mustard or sweet and sour sauce | 30 | 15-45 | 117 |
| 121. Dips | such as legume or dairy-based | 30 | 15-45 | 92 |
| 122. Major main entree sauce | such as spaghetti sauce | 125 | 100-200 | 145 |
| 123. Minor main entree sauce | such as pizza sauce, pesto sauce, or other sauces used as toppings | 60 | 50-100 | 102 |
| 124. Major condiments | such as ketchup, steak sauce, soy sauce, vinegar, teriyaki sacue or marinades | 15 | 10 – 20 | 100 |
| 125. Minor condiments | such as horseradish, hot sauce, mustard, or Worcestershire sauce | 5 | 5 – 10 | 48 |
| **Snacks** |  |  |  |  |
| 126. Chips, pretzels, popcorn, extruded snacks | + grain-based snack mixes and fruit-based snacks, such as fruit chips | 50 | 40-60 | 375 |
| 127. Nuts or seeds for use as snacks |  | 50 | 40-60 | 88 |
| **Sugars and Sweets** |  |  |  |  |
| 137. Jams, jellies, marmalades etc. | + fruit butters and spreads | 15 | 10 – 20 | 145 |
| 141. syrups | including chocolate, maple and corn syrup | 60 | 30-60 | 50 |
| **Vegetables** |  |  |  |  |
| 150. Pickles |  | 30 | N/A | 54 |
